# Data on taxonomic annotation and diversity of 18S rRNA gene amplicon libraries derived from high throughput sequencing

**DOI:** 10.1016/j.dib.2019.104213

**Published:** 2019-07-02

**Authors:** Takafumi Kataoka, Ryuji Kondo

**Affiliations:** Department of Marine Science and Technology, Fukui Prefectural University, Obama, Fukui, 917-0003, Japan

**Keywords:** Protists, 18S rRNA gene, High throughput sequencing (HTS), MiSeq, V4–V5 hypervariable region

## Abstract

This Data in Brief article is a supporting information for the research article entitled “Protistan community composition in anoxic sediments from three salinity-disparate Japanese lakes” by Kataoka and Kondo (2019) [1]. Summary of 18S rRNA gene sequences originated from anoxic sediment of three lakes in two seasons using high throughput sequencing techniques (MiSeq, Illumina) was shown in this data article. Supergroup-level taxonomy was compared between the SILVA search for SILVA database and BLASTn search for the PR2 database. Alpha diversity was calculated in each sample, and beta-diversity was calculated among the six amplicon libraries. Partial sequence length between the primer set of 574*f and 1132R Hugerth et al., 2015 was compared between the forward read and the combined read.

**Table:** Specifications table

Subject area	*Biology*
More specific subject area	*Microbial Ecology*
Type of data	*Tables, figures, FASTQ*
How data was acquired	*High throughput sequencing data of 18S rRNA gene amplicon using Illumina MiSeq sequencing*
Data format	*Raw and analysed*
Experimental factors	*Genomic DNA was extracted from anoxic sediment in lakes.*
Experimental features	Amplicon was generated using a primer set of 574*f and 1142R.
Data source location	*Lakes Hiruga and Suigetsu in Mikata Lake Group in Fukui Prefecture and Lake Biwa in Shiga Prefecture, Japan.*
Data accessibility	*Analysed data is presented in the article. Raw DNA sequences are available in the DNA Data Bank of Japan (DDBJ) under the accession numberDRA007713(**https://ddbj.nig.ac.jp/DRASearch/submission?acc=DRA007713**).*
Related research article	*T. Kataoka, R. Kondo. Protistan community composition in anoxic sediments from three salinity-disparate Japanese lakes. Estuarine, Coastal and Shelf Science, 224, 34–42 (2019).**https://doi.org/10.1016/j.ecss.2019.04.046*

**Table:** 

**Value of the data** • Comparing methods of annotating taxonomic path for 18S rRNA gene sequence is valuable because sequence in public database is still insufficient for identifying diverse eukaryotic microbes. • Information of partial sequence length between the forward- and reverse-primer is valuable for understanding protistan composition in natural environment where unknown microbes inhabit. • Alpha and beta diversities of protistan genotypes in lacustrine sediments are rare example.

## 1 Data

**Fig. 1:**
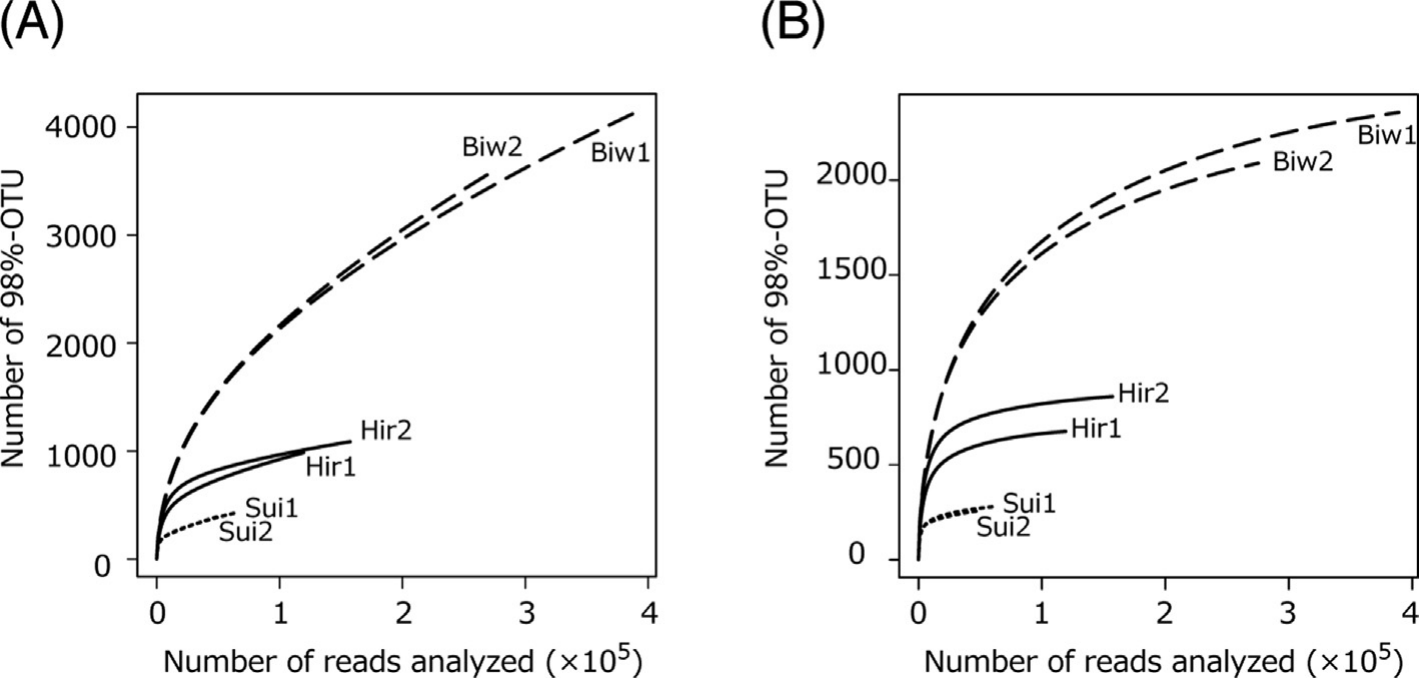
Rarefaction curves of 98% similarity-based-OTUs in each sample (A) including all reads and (B) with singleton reads removed.

**Fig. 2:**
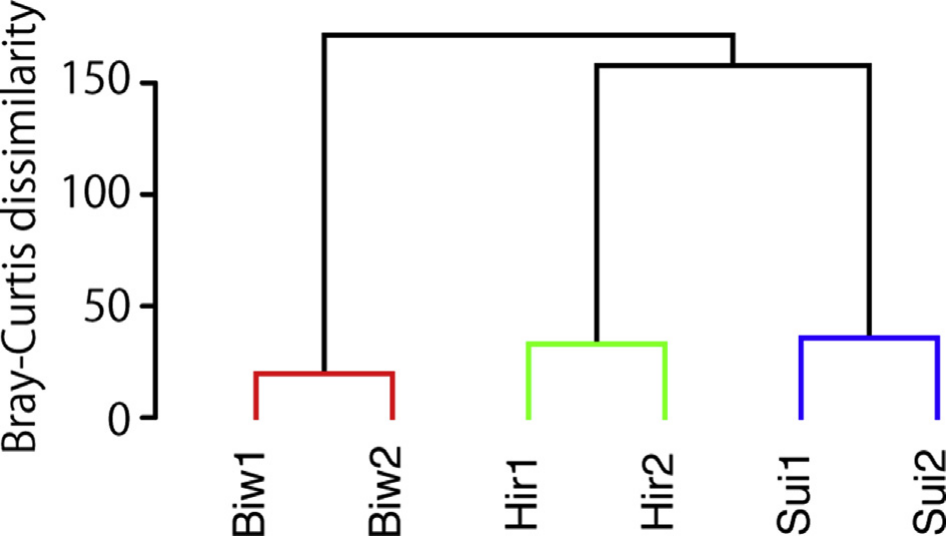
Similarity profile analysis to detect significant clusters (p < 0.05). Dissimilarity was calculated by relative abundance data of sequence reads using the Bray-Curtis index, and significantly distant samples were clustered using Ward's method.

**Fig. 3:**
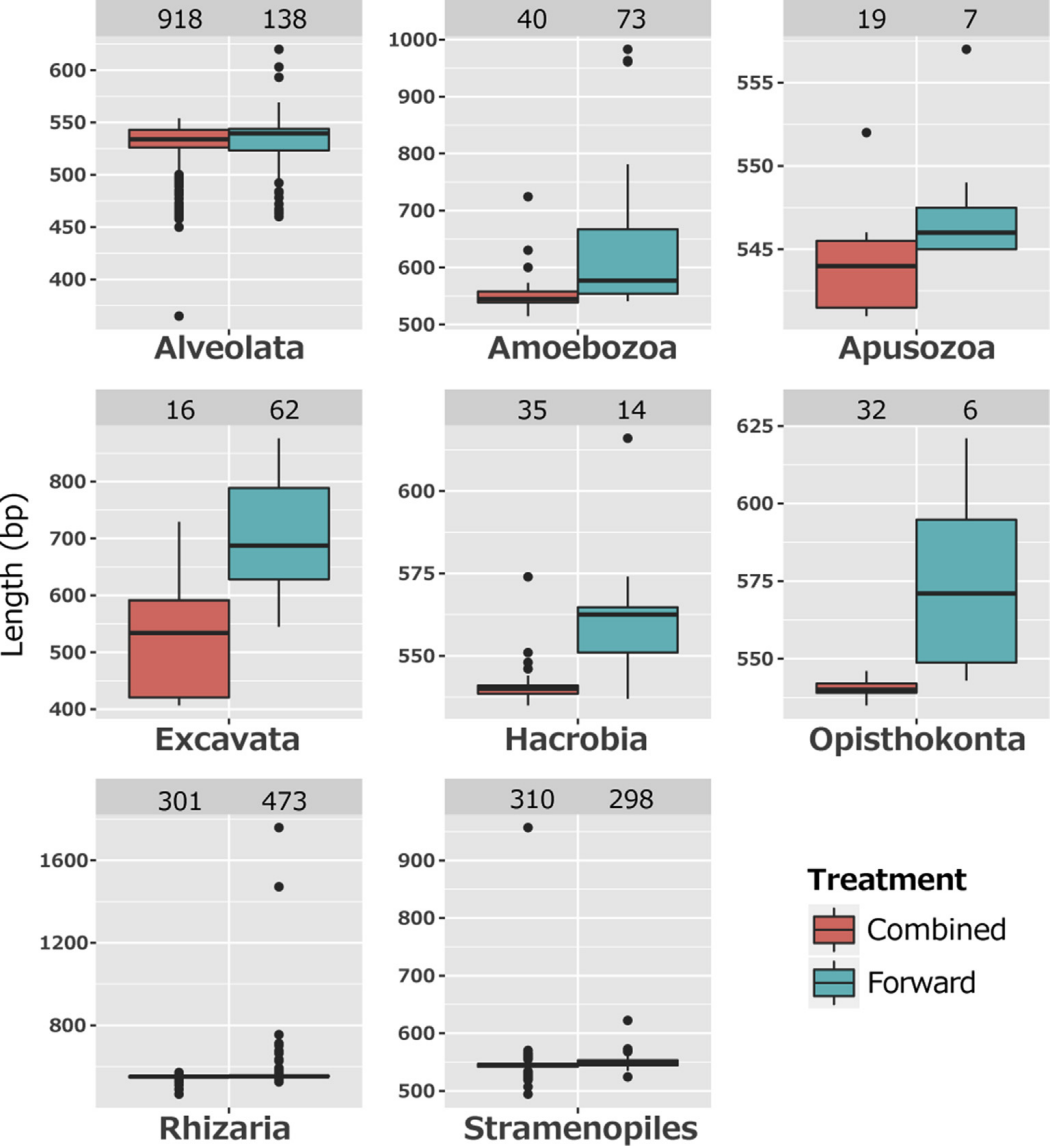
Partial sequence length between the primer sets, 574*f and 1132R [2], of sequences in the PR2 database to which OTU representatives received the best hit using a BLAST search. The labels Combined and Forward indicate the combined sequences yielded from both primers and single sequences yielded from the forward primer, respectively. The number on the top of each plot shows the number of sequences analysed. The bar in the box indicates the median value. The top and bottom of the boxes indicate the upper and lower quartiles, respectively.

Raw read from MiSeq was quality controlled and grouped into OTUs at 98% sequence similarity level, then OTUs that is constructed only one sequence (singleton) was removed (Table 1). Annotation method for taxonomic path for representative sequence of each OTU of 18S rRNA gene sequence was compared in order to clarify suitable method for identifying supergroup taxonomy (Table 2). Alpha diversity was compared by calculating rarefaction curve (Fig. 1) in each sample, and beta diversity was determined by calculating by similarity profile analysis of all samples (Fig. 2). Partial sequence length between the forward and reverse primers was compared between independently generated query sequences (Fig. 3).

**Table 1:** Summary of sequence read and OTU number before and after singleton was removed.

	Hiruga1	Hiruga2	Suigetsu1	Suigetsu2	Biwa1	Biwa2
Including all reads						
Sequence read	119529	157402	63764	48948	390826	276815
OTU	984	1086	426	391	4141	3612
After removed singleton						
Sequence read	119221	157176	63619	48815	389041	275292
OTU	676	860	281	258	2356	2089
Number of singleton	308	226	145	133	1785	1523
% singleton	31.3	20.8	34.0	34.0	43.1	42.2

**Table 2:** Number of OTUs showing mismatch between a SINA search (the SILVA database ver. 132) and a BLASTn search (the PR2 database ver. 4.10.0) identification at supergroup taxonomy.

		Number of OTUs	SINA × SILVA identification											
			Alveolata	Amoebozoa	Archaeplastida	Opisthokonta	Rhizaria	Stramenopiles	Picozoa	Centrohelida	Cryptophyceae	Haptophyta	IncertaeSedis	NAMAKO-1
BLASTn × PR2 identification	Alveolata	62	–		12	10	2	38						
	Amoebozoa	22		–									20	1
	Archaeplastida	42	25	5	–	4		5			1	2	20	
	Opisthokonta	138	76	1	13	–	4	18					12	
	Rhizaria	10	5			1	–	1				3		
	Stramenopiles	57	45		5	3	4	–						
	Hacrobia	113				4		1	2	2	11	73	20	
	Apusozoa	29											29	
	Unknown	3		2				1

## 2 Experimental design, materials, and methods

Lacustrine sediments were collected from the southern basin of Lake Biwa, and the central basins of Lake Suigetsu and Lake Hiruga using an Ekman–Birge-type bottom sampler (RIGO, Saitama, Japan) [1]. Surface sediment was subsampled from the 0–5 cm depth using a syringe with the needle-end cut-off. Total nucleic acids were extracted from the 0.5 g sediment samples using a FastDNA Spin Kit for Soil (MP Biomedicals, LLC, Solon, OH) according to the manufacturers' instructions. An amplicon library for high throughput sequencing analysis of protists 18S rRNA genes was constructed using a primer set targeting to the V4–V5 hypervariable region in protist 18S rRNA genes named 574*f (5′-CGGTAAYTCCAGCTCYV-3′) and 1132R (5′-CCGTCAATTHCTTYAART-3′) [2]. PCR amplification was performed in a 25 μL reaction mixture containing 1 × KAPA HiFi HotStart ReadyMix (KAPA Biosystems), 0.3 μM of each primer and 3 μL of ten-times diluted gDNA that corresponded to 0.4–1.3 ng of gDNA, under cycling conditions as follows: heating to 94 °C for 3 min to activate the hot-start DNA polymerase, 30 cycles at 94 °C for 30 s, annealing at 51 °C for 30 s, elongation at 72 °C for 45 s, then a final elongation at 72 °C for 7 min. Amplicon with expected lengths of 560 bp, which was determined using agarose gel electrophoresis, were purified and labelled with an index primer set attaching to both the 5′ and 3′ ends (NEBNext Multiplex Oligos, New England BioLabs), then sequenced using MiSeq Reagent kit v3 for 2 × 300 bp (Illumina, CA, USA). All of the generated sequence reads were de-multiplexed according to the index primers and processed using the software package Claident ver. 0.2.2017.07.26 [3], as previously described with a minor modification [4]. For generating the pared-end sequences, forward and reverse reads were combined with >50 bp overlapping ends of each read by VSEARCH. The combined reads of >400 bp length with a quality value of >30 were used for establishing operational taxonomic units (OTUs) using a 98% cut-off level. The OTUs that were detected as a single read within all samples (singletons) were omitted because too many singletons, which accounted for 21%–43% of OTUs (Table 1). A representative sequence of each OTU was filtered to split the sequences into ribosomal RNA (rRNA) and non-rRNA genes using riboPicker [5], and both rRNA and non-rRNA sequences were identified using the SINA programme [6] with reference to the SILVA database (SSURef_NR99_132 [7]). The taxonomic path for both rRNA and non-rRNA sequences was also obtained from the top hit of a BLASTn search [8], with reference to the PR2 database (ver. 4.10.0 [9]). A given p-value cut-off of 1 × 10^−50^ was used to remove non-rRNA genes [10]. In order to focus on potentially heterotrophic protists, fungal and autotrophic sequences were removed according to the PR2 taxonomy path. Rarefaction curves were calculated using the vegan package, ver. 2.4 [11]. Similarity profile analysis was conducted using the clustsig package, ver. 1.1. The dissimilarity was calculated by relative abundance data of sequence reads using the Bray-Curtis index, and significantly distant samples were clustered using Ward's method. All statistical analyses were conducted using R software ver. 3.3.2 (http://cran.r-project.org).

## References

[1] Kataoka T., Kondo R. (2019). Protistan community composition in anoxic sediments from three salinity-disparate Japanese lakes. Estuarine. Coastal and Shelf Science.

[2] Hugerth L.W., Muller E.E.L., Hu Y.O.O., Lebrun L.A.M., Roume H. (2015). Systematic Design of 18S rRNA Gene Primers for Determining Eukaryotic Diversity in Microbial Consortia (vol 9, e95567, 2014). PLoS One.

[3] Tanabe A.S., Toju H. (2013). Two new computational methods for universal DNA barcoding: a benchmark using barcode sequences of bacteria, archaea, animals, fungi, and land plants. PLoS One.

[4] Kataoka T., Yamaguchi H., Sato M., Watanabe T., Taniuchi Y., Kuwata A., Kawachi M. (2017). Seasonal and geographical distribution of near-surface small photosynthetic eukaryotes in the western North Pacific determined by pyrosequencing of 18S rDNA. FEMS Microbiol. Ecol..

[5] Schmieder R., Lim Y.W., Edwards R. (2012). Identification and removal of ribosomal RNA sequences from metatranscriptomes. Bioinformatics.

[6] Pruesse E., Peplies J., Glöckner F.O. (2012). SINA: accurate high-throughput multiple sequence alignment of ribosomal RNA genes. Bioinformatics.

[7] Quast C., Pruesse E., Yilmaz P., Gerken J., Schweer T., Yarza P., Peplies J., Glöckner F.O. (2013). The SILVA ribosomal RNA gene database project: improved data processing and web-based tools. Nucleic Acids Res..

[8] Johnson M., Zaretskaya I., Raytselis Y., Merezhuk Y., McGinnis S., Madden T.L. (2008). NCBI BLAST: a better web interface. Nucleic Acids Res..

[9] Guillou L., Bachar D., Audic S., Bass D., Berney C., Bittner L., Boutte C., Burgaud G., de Vargas C., Decelle J., del Campo J., Dolan J.R., Dunthorn M., Edvardsen B., Holzmann M., Kooistra W.H.C.F., Lara E., Le Bescot N., Logares R., Mahé F., Massana R., Montresor M., Morard R., Not F., Pawlowski J., Probert I., Sauvadet A.L., Siano R., Stoeck T., Vaulot D., Zimmermann P., Christen R. (2013). The Protist Ribosomal Reference database (PR2): a catalog of unicellular eukaryote Small Sub-Unit rRNA sequences with curated taxonomy. Nucleic Acids Res..

[10] Chervitz S.A., Aravind L., Sherlock G., Ball C.A., Koonin E.V., Dwight S.S., Harris M.A., Dolinski K., Mohr S., Smith T., Weng S., Cherry J.M., Botstein D. (1998). Comparison of the complete protein sets of worm and yeast: orthology and divergence. Science.

[11] Oksanen J., Kindt R., Legender P., O'Hara B., Simpson G.L., Stevens M.H.H., Wagner H. (2008). Vegan: Community Ecology Package.

